# A Limited Role for PI(3,4,5)P_3_ Regulation in Controlling Skeletal Muscle Mass in Response to Resistance Exercise

**DOI:** 10.1371/journal.pone.0011624

**Published:** 2010-07-16

**Authors:** D. Lee Hamilton, Andrew Philp, Matthew G. MacKenzie, Keith Baar

**Affiliations:** Division of Molecular Physiology, University of Dundee, Dundee, Scotland, United Kingdom; McMaster University, Canada

## Abstract

**Background:**

Since activation of the PI3K/(protein kinase B; PKB/akt) pathway has been shown to alter muscle mass and growth, the aim of this study was to determine whether resistance exercise increased insulin like growth factor (IGF) I/phosphoinositide 3-kinase (PI3K) signalling and whether altering PI(3,4,5)P_3_ metabolism genetically would increase load induced muscle growth.

**Methodology/Principal Findings:**

Acute and chronic resistance exercise in wild type and muscle specific PTEN knockout mice were used to address the role of PI(3,4,5)P_3_ regulation in the development of skeletal muscle hypertrophy. Acute resistance exercise did not increase either IGF-1 receptor phosphorylation or IRS1/2 associated p85. Since insulin/IGF signalling to PI3K was unchanged, we next sought to determine whether inactivation of PTEN played a role in load-induced muscle growth. Muscle specific knockout of PTEN resulted in small but significant increases in heart (PTEN^+/+^  = 5.00±0.02 mg/g, PTEN^−/−^  = 5.50±0.09 mg/g), and TA (PTEN^+/+^  = 1.74±0.04 mg/g, PTEN^−/−^  = 1.89 ±0.03) muscle mass, while the GTN, SOL, EDL and PLN remain unchanged. Following ablation, hypertrophy of the PLN, SOL or EDL muscles was similar between PTEN^−/−^ and PTEN^+/+^ animals. Even though there were some changes in overload-induced PKB and S6K1 phosphorylation, 1 hr following acute resistance exercise there was no difference in the phosphorylation state of S6K1 Thr389 between genotypes.

**Conclusions/Significance:**

These data suggest that physiological loading does not lead to the enhanced activation of the PI3K/PKB/mTORC1 axis and that neither PI3K activation nor PTEN, and by extension PI(3,4,5)P_3_ levels, play a significant role in adult skeletal muscle growth.

## Introduction

The development of skeletal muscle mass and function is dependent upon signalling through the insulin like growth factor (IGF)/protein kinase B (PKB/akt)/mammalian target of rapamycin complex 1 (mTORC1) axis [1], [2], [3]. Deletion or inhibition of components of this axis leads to smaller muscles [4], [5] and impaired hypertrophy in response to loading [6], regeneration [7] and growth factor stimulation [5], [8], [9]. In contrast, over-expression of various components of this pathway leads to muscle growth [7], [10], [11] and protection from atrophy [6], [12], [13].

Growth factors, such as IGF-1, activate receptor tyrosine kinases leading to the membrane recruitment and activation of the class 1_a_ phosphatidylinositol 3-kinase (PI3K) [14], [15]. At the membrane, PI3K phosphorylates PI(4,5)P_2_ at the D3 position on the inositol ring producing PI(3,4,5)P_3_ [16], an important second messenger required for the oncogenic [17] and growth factor [18] induced activation of mTORC1. PI(3,4,5)P_3_ activates mTORC1 through the recruitment and activation of PKB [19]. PKB is an important cellular mediator of growth factor signalling with cellular roles including regulation of cell survival, growth, proliferation, angiogenesis and metabolism [20]. A clear role for PKB in controlling skeletal muscle mass has been defined with a range of genetic techniques combined with *in vivo* and *in vitro* studies. Electroporating constitutively active PKB in to adult rat skeletal muscle increases muscle mass and protects against unloading/denervation induced atrophy [6], [7], overexpressing constitutively active PKB in C_2_C_12_ myotubes increases myotube diameter [8] and in both instances these effects are antagonised by PI(3,4,5)P_3_ phosphatases [6], [8].

In skeletal muscle, enhanced PI3K expression or downstream signalling promotes myoblast differentiation [21] and skeletal muscle growth [6], [7] but does not result in transformation of terminally differentiated skeletal muscle [22], [23]. Due to the growth promoting effects of PI(3,4,5)P_3_, the amount of this phospholipid is tightly controlled by several lipid phosphatases including the SH2-containing 5′-inositol phosphatase (SHIP) 2 [16] and PTEN. Whereas SHIP2 removes the 5-phosphate, PTEN is the only enzyme capable of directly antagonising PI3K by dephosphorylating the D3 phosphate of PI(3,4,5)P_3_ [24], [25].

As with PI3K, PTEN plays an important role in many cellular functions such as cell migration [26], PDGF induced membrane ruffling [27], IGF-1/insulin sensitivity [28] and oncogenesis [29]. In many instances loss of PTEN leads to enhanced tumour formation [30]. However, loss of PTEN in striated muscle does not lead to transformation [22], [23]. Interestingly, striated muscle specific PTEN null mice have been reported to have a ∼50% increase in heart mass [22] and are protected from high fat diet induced insulin resistance and diabetes [23] even though at baseline they showed a slight reduction in insulin stimulated PKB Thr^308^ phosphorylation [23].

Since the activation of the PI3K signalling pathway leads to muscle growth, the aim of the current work was to determine whether growth factor signalling through PI3K was induced by resistance exercise and whether genetic deletion of PTEN would enhance the hypertrophic response of muscle to overload and acute resistance exercise. We hypothesised that muscle specific loss of PTEN would lead to blunted insulin stimulated PKB phosphorylation but enhanced muscle hypertrophy and hypertrophic signalling following synergist ablation and acute resistance exercise.

## Methods

### Materials

Rabbit anti-S6K1 was obtained from Santa Cruz Biotechnology (California, USA), rabbit-anti: p-S6K1^T389^, p-PKB^T308^, t-PKB, were from Cell Signalling Technologies (Massachusetts, USA). All other chemicals were from Sigma-Aldrich unless stated otherwise.

### Animals

All procedures were approved by the University of Dundee research ethics committee and performed under UK Home Office project licence number 60/3441. Surgical and collection procedures on transgenic mice ∼20–35 g took place under inhaled anaesthetic using a 2.5% concentration of isoflurane. Animals were allowed to recover for the appropriate time period post stimulation and were terminated after muscle collection under anaesthesia.

### Generation of Muscle Specific PTEN null mice

The generation of MCK-Cre mice and PTEN Floxed mice have previously been described [31], [32]. PTEN flox/flox mice were a kind gift from Professor Doreen Cantrell University of Dundee and the MCK-Cre^+/−^ mice were a kind gift from Professor Dario Alessi University of Dundee. The MCK-Cre and PTEN flox/flox mice were mated and the subsequent offspring were then used as breeders to generate MCK-Cre controls termed PTEN+/+ (PTEN+/+ MCK-Cre+/−), muscle specific PTEN null mice termed PTEN−/− (PTEN flox/flox MCK-Cre+/−), PTEN flox controls (PTEN flox/flox MCK-Cre−/−) and wild types (PTEN+/+ MCK-Cre−/−). All genotyping was performed by PCR using genomic DNA isolated from the tail tip of 3-to-4-week-old mice. Genotyping was then repeated on skeletal muscle DNA from experimental animals to confirm that experimental animals were of the correct genotype. The primers for identifying carriers of the MCK-Cre transgene (5′-ATGTCCAATTTACTGACCG-3′ and 5′-CGCCGCATAACCAGTGAAAC-3′) were used under the following conditions: 1 cycle of 94°C for 2 min, 39 cycles of 92°C for 30 s, 50°C for 30 s, and 72°C for 45 s followed by one cycle of 72°C for 10 min. The primers for identifying carriers of the PTEN flox transgene (5′- GCCTTACCTAGTAAAGCAAG -3′ and 5′- GGC AAA GAA TCT TGG TGT TAC -3′) were used under the following conditions: 1 cycle of 94°C for 3 min 35 cycles of 94°C for 30 s, 53°C for 40 s, and 72°C for 35 s followed by one cycle of 72°C for 10 min. PCR products were then subjected to electrophoresis on a 2% agarose gel and genotype was established by the presence of a 500 bp band from the MCK-Cre reaction and either a 200 bp product for PTEN−/− animals and a 250 bp product for PTEN+/+ animals with a double band for heterozygotes (Figure S1).

### Acute Electrical Stimulation

The resistance exercise model was performed as described previously [33]. Tetanic contractions were elicited using a Grass stimulator at a frequency of 100 Hz, 6–12 V, 1 ms duration, 9 ms delay for 10 sets of 6 repetitions. Each repetition lasted 2 s, a 10 s recovery was permitted between repetitions, and a 1 min recovery was allowed between sets of 6 repetitions resulting in a 20 min stimulation protocol. Following the acute bout of contractions, stimulated and contralateral muscles were rapidly removed, snap frozen in liquid nitrogen, and stored at −80°C until processed.

### Load Induced Hypertrophy

In order to study the overload induced growth response of skeletal muscle from muscle specific PTEN null mice three different muscles were overloaded. The plantaris (PLN) and soleus (SOL) muscles were overloaded by removal of the gastrocnemius (GTN) muscle and in a separate group of animals the extensor digitorum longus (EDL) muscle was overloaded by removal of the tibialis anterior muscle. The different models were chosen as models of continuous overload (PLN and SOL) with (PLN) or without (SOL) changes in recruitment and an intermittent overload model (EDL). The continuous and intermittent models were used in case the loss of PTEN resulted in a prolonged signal for muscle growth that was obscured in the continuous overload paradigm. After a pilot study, the following protocol was established: ablation of the GTN for 10 days to induce hypertrophy of the PLN and SOL; and in a separate group of animals ablation of the TA for 4 weeks to induce hypertrophy in the EDL. The synergist ablation model was performed as described previously [34]. Briefly animals were anaesthetised and the area above the incision shaved and sterilised. The GTN was isolated and severed at the Achilles tendon and the distal two thirds of the GTN removed leaving the PLN and SOL muscles and their blood and nerve supply intact. A similar procedure was carried out for removal of the tibialis anterior (TA) except the TA was removed in totem whilst leaving the EDL intact. The overlying skin and fascia were sutured separately and the animal was then moved to a temperature-controlled environment to recover. All animals returned to normal activity within one hour. In the recovery period prior to collection animals were monitored on a daily basis for signs of pain or post-operative infection. No signs of discomfort or distress were noted. On the day of collection, animals were anaesthetised and overloaded and contralateral control muscles were rapidly removed rinsed in PBS, all connective/scar tissue was removed using a dissection microscope, then the muscles were snap frozen in liquid nitrogen, and stored at −80°C until processed. Following collection animals were terminated by cervical dislocation.

### Protein Extraction and quantification

Muscles were rapidly removed and rinsed to remove blood and fur. Tissue was snap frozen in liquid nitrogen and stored at −80°C. For processing, muscle was powdered on dry ice using a mortar and pestle and polytron homogenised in 10-fold mass excess of ice cold sucrose lysis buffer (50 mM Tris pH 7.5, 250 mM Sucrose, 1 mM EDTA, 1 mM EGTA, 1% Triton X 100, 50 mM NaF, 1 mM NaVO_4_ Na_2_(PO_4_)_2_ and 0.1% DTT). This was briefly vortexed and centrifuged at 4°C for 10 mins at 11,000 rpm to remove insoluble material. Protein concentrations were determined using the DC protein assay (Bio-Rad, Hercules; California, USA).

### Western Blotting

Equal aliquots of protein were diluted in Laemmli sample buffer and boiled for 5 mins. 5–10 µg of sample was then subjected to SDS-PAGE on 10% acrylamide gels at a constant current equal to 20 mA per gel and transferred to Protran nitrocellulose membrane (Whatman; Dassel, Germany) using a BioRad semidry transfer apparatus at 100 V for 1 hour. Membranes were blocked in 5% dry milk in TBS-T and then incubated over night at 4°C with appropriate primary antibody in TBST at 1∶1000. The membranes were then washed 3x in TBST before incubation for 1 hour at room temperature with peroxidase-conjugated secondary antibodies in TBST at 1∶10000 (Perbio Science; Cramlington, UK). Antibody binding was detected using an enhanced chemiluminescence HRP substrate detection kit (Millipore; Watford, UK). Imaging and band quantification were carried out using a Chemi Genius Bioimaging Gel Doc System (Syngene; Cambridge, UK).

### Immunocytochemistry

PLN muscles were rapidly dissected, rinsed in PBS and blotted dry before being mounted in Tissue Tek OCT compound (Sakura Finetek, Japan) and frozen in liquid nitrogen cooled isopentane. Tissues were sectioned 8 µm thick at −20°C onto glass coverslips. Coverslips were then rinsed 3 times in PBS and fixed in 4% PFA-PBS, aldehydes were quenched in 100 mM glycine for 15 mins before rinsing 3 times in 0.2% fish skin gelatine (made in PBS). Coverslips were then blocked in Goat Serum (1∶10, 0.2% FSG; Jackson ImmunoResearch Europe; Suffolk, UK) for 10 mins, and rinsed once in 0.2% FSG before being incubated in rabbit anti-PTEN (1∶100), for 20 mins at room temperature. Primary was rinsed 3 times with 0.2% FSG before being placed in secondary [goat anti-rabbit Alexa Fluor 594 (Invitrogen; California, USA) at 1∶500, with DAPI at 1∶1000 using an 10 µg/µl stock]. Secondary was rinsed off 3 times with 0.2% FSG and 3 times in MiliQ water before being blotted dry and mounted in Vectashield® Mounting Medium (Vector Laboratories; California, USA). Sections were analyzed on a Deltavision DV3 wide field deconvolution microscope.

### Muscle Fibre Isolation

Muscle fibres were isolated using a protocol described previously [35], [36]. Briefly, 6 PLN muscles from each experimental group were grouped and incubated for 4 h at 37°C in a culture medium containing minimum essential medium with Earle's salt and L-glutamine (GIBCO; California, USA) supplemented with 10% fetal bovine serum (GIBCO; California, USA), 0.2% type I collagenase, 100 U/ml penicillin, and 100 µg/ml streptomycin (GIBCO; California, USA). Fibers were separated from connective tissue by fine tweezers under a dissecting microscope and subjected to tissue lysis as previously described.

### Insulin Responsiveness

Animals were anaesthetised in groups of 3 as previously described and maintained in an anaesthetic chamber. Fast acting human insulin (Actrapid, Novo Nordisk; Bagsvaerd, Denmark) (0.1, 1, 10, 100, or 1000 mU/g), diluted in sterile 0.2% BSA-PBS, was injected intraperitoneally into an animal. Controls were injected with an equal volume of 0.2% BSA-PBS. The concentration of each dose was adjusted such that each animal was injected with 10 µl/g body weight. Following 10 mins of insulin stimulation, animals were terminated by cervical dislocation and EDL and SOL muscles were rapidly removed, rinsed in PBS and snap frozen in liquid nitrogen before being stored at −80°C.

### Statistics

Data are given as mean ± SEM values with a minimum n of 3. Statistical significance (p<0.05 unless otherwise stated) of the data was determined by Student's T-test or a single factor ANOVA with posthoc analysis using Tukey's honestly significant difference test.

## Results

### Resistance exercise does not increase IGF-1 receptor phosphorylation

**Figure 1:**
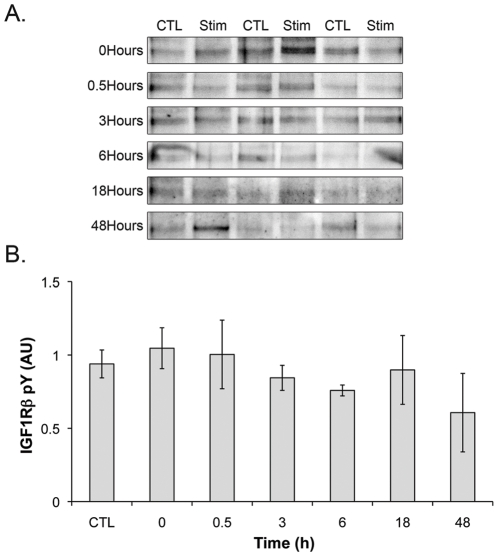
The IGF-1 receptor is not tyrosine phosphorylated following a single bout of resistance exercise. immunoblots. The IGF-1R was immunoprecipitated from the right and left TA muscles (n = 3) following resistance exercise and the resulting western blot was probed with the pY20 phosphotyrosine antibody (A) and the immunoreactivity was quantified in panel (B).

Since the anabolic affects of insulin and IGF-1 are mediated through the IGF-1R, we determined the tyrosine phosphorylation of the IGF-1R following uniaxial resistance exercise by immunoprecipitating the receptor and determining phosphotyrosine content by western blot. At no time point following acute resistance exercise was there an increase in IGF-1R tyrosine phosphorylation (Figure 1), suggesting that the IGF-1R is not activated by acute resistance exercise.

### Resistance exercise does not increase IRS1/2 and pY associated p85

**Figure 2:**
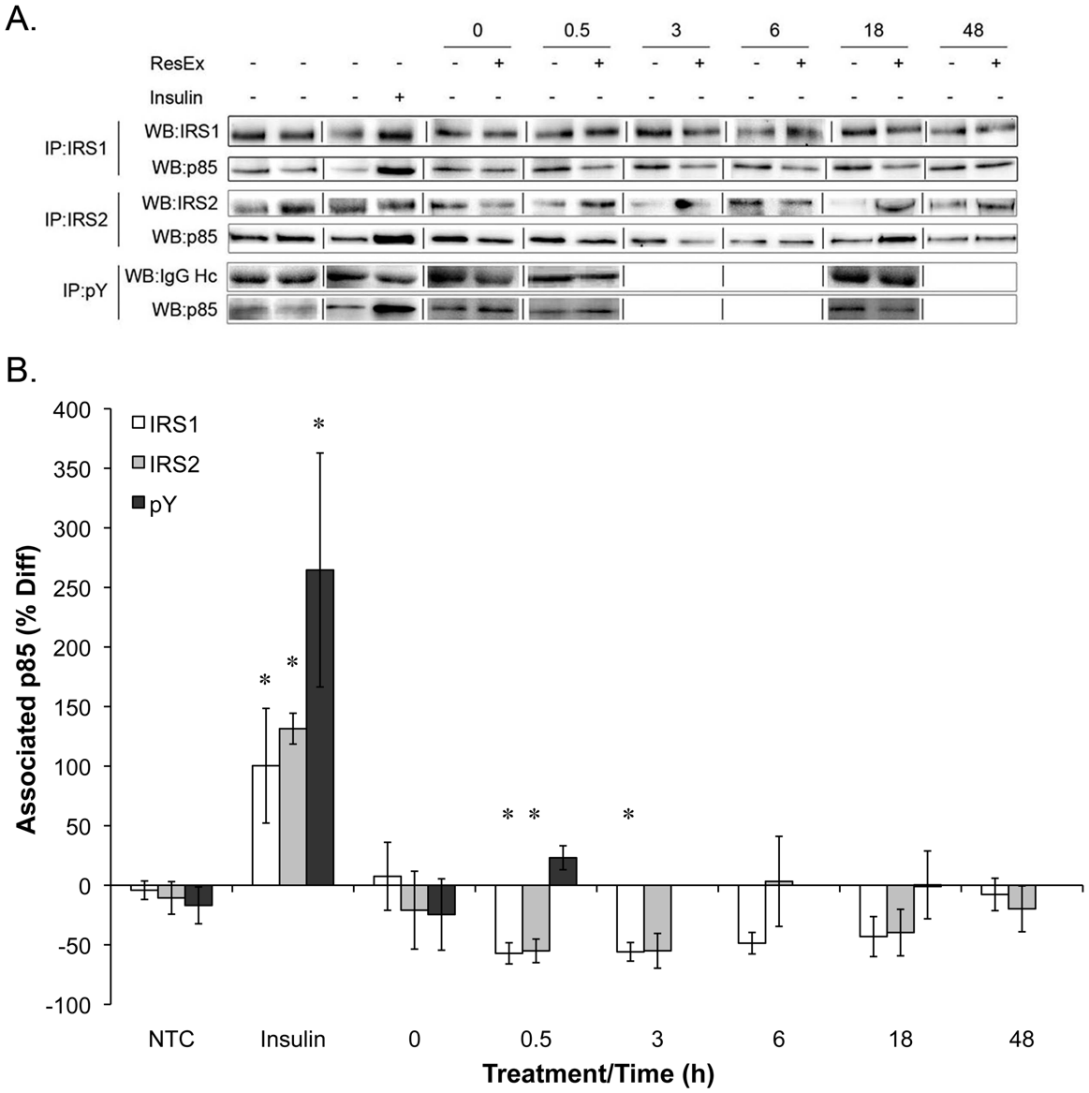
IRS1/2 and pY associated p85 is unchanged following a single bout of resistance exercise. IRS1, IRS2, or phosphotyrosine containing proteins were immunoprecipitated from the right and left TA muscles (n = 4) following resistance exercise and the resulting western blot (A) was probed with the antibody to the p85 regulatory subunit of PI3K and the immunoreactivity was quantified in panel (B). * Indicates significantly different from control leg.

The second step in the activation of mTORC1 by growth factors is the recruitment of IRS1/2 and PI3K to the receptor. Therefore, we next sought to determine whether there was a change in IRS1/2 associated p85 after resistance exercise. Insulin potently increased the amount of IRS1/2 associated p85 (Figure 2). However, at no time after resistance exercise was there an increase in IRS1/2 associated p85 (Figure 2). In fact, IRS1 associated p85 was significantly reduced at 0.5 hr (−57.1±8.9%) and 3 hr (−55.9±7.8%) and IRS2 associated p85 was significantly reduced at 0.5 hr (−55.1±9.9%). There was also no significant change in phosphotyrosine-associated p85 at 0 hr, 0.5 hrs or 18 hrs post contraction (Figure 2).

### Genotyping and PTEN Tissue Distribution

Since PI3K was not activated following resistance exercise, we next sought to determine whether this pathway was increased due to inactivation of the 3-phosphatase PTEN. Muscle specific PTEN null mice were confirmed by the presence of the 500 bp MCK-Cre PCR product in conjunction with a single 250 bp product from the PTEN flox PCR reaction, while MCK-Cre controls were confirmed by the presence of a 200 bp product from the Cre PCR reaction (Figure S1). As expected from the wide range of tissues affected by germline PTEN mutations [37], [38], [39], PTEN was highly expressed in all tissues analysed with the exception of skeletal muscle (Figure S1). Surprisingly PTEN^−/−^ mice did not demonstrate a complete knockout in skeletal or cardiac muscle, directly contrasting previously published observations [22], [23]. To confirm muscle specific knockout of PTEN we analysed the expression of PTEN in whole brain/kidney/PLN lysates, isolated thymocytes and isolated PLN muscle fibres. As a positive control for the function of the Cre-loxP system in our line we also ran lysates from isolated thymocytes expressing Cre under the control of the LCK (thymocyte-specific) promoter. Again, PTEN expression in skeletal muscle was significantly lower than brain, kidney, or isolated thymocytes. As described above, there was a partial knockout of PTEN in the whole PLN muscle, however the remaining level of PTEN was higher than in the PTEN^−/−^ thymocytes. In the isolated muscle fibres, expression of PTEN was extremely low in the PTEN^+/+^ animals and was reduced in the PTEN^−/−^ animals to the same extent as seen in the PTEN^−/−^ thymocytes (Figure S1). We next determined the expression of PTEN in skeletal muscle using immunohistochemistry. Again, we were unable to detect significant amounts of PTEN in skeletal muscle fibres of either PTEN^+/+^ or PTEN^−/−^ animals. PTEN positive areas were seen between muscle fibres in both PTEN^+/+^ and PTEN^−/−^ muscles, suggesting that PTEN was largely restricted to cells of endothelial origin. This suggests that the PTEN detected by western blot in the PTEN^−/−^ muscle is from vascular endothelial, neural and blood cells all of which are rich in PTEN.

### Skeletal and Cardiac Muscle Mass

**Figure 3:**
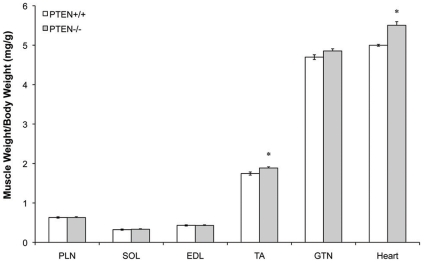
Alterations in striated muscle mass in PTEN knockout mice. The mass of select striated muscles relative to body mass was determined for PTEN^+/+^ (n = 19) and PTEN^−/−^ mice (n = 20). * Indicates significantly different from wild type (p<0.05).

To assess the effect of PTEN ablation on skeletal and cardiac muscle mass we determined the wet mass of a wide range of skeletal muscles and the heart of a large number of animals [PTEN^−/−^ n = 20 (10 male +10 female), PTEN^+/+^ n = 19 (9 male +10 female)] (Figure 3). Similar to previous observations [23] the mass of the GTN, SOL, EDL, and PLN muscles were unaffected by the loss of PTEN (Figure 3). However, the TA [(8%) PTEN^+/+^  = 1.74±0.04 mg/g, PTEN^−/−^  = 1.89±0.03 (p<0.05)] muscles were significantly larger in the PTEN^−/−^ mice. As has been reported, the heart mass was increased (Figure 3), though the magnitude of the effect was less (∼10%) than seen previously (∼50%); [22].

### Insulin Responsiveness

Several reports have demonstrated a loss of insulin sensitivity following PTEN ablation [23], [28]. To confirm PTEN knockout in our mice, a dose response curve for insulin stimulated PKB Thr^308^ phosphorylation was performed (Figure S2). In the SOL muscle, there was no effect of PTEN ablation on insulin stimulated PKB Thr^308^ phosphorylation (Figure S2). However, although the maximal response of the EDL was unaltered, there was a right shift in the curve suggesting impaired insulin-stimulated phosphorylation of PKB (Figure S2).

### Muscle Growth Response Following Ablation

**Figure 4:**
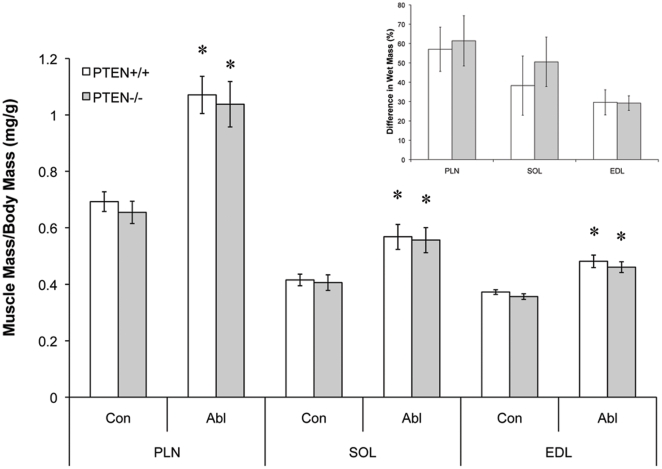
PTEN knockout in striated muscle does not alter muscle hypertrophy in response to overload. The plantaris (PLN) and soleus (SOL) muscles were overloaded by ablation of the gastrocnemius muscle for 10 days. In a separate group of animals the extensor digitorum longus (EDL) muscle was overloaded by removal of the tibialis anterior muscle for 4 weeks. Data presented are mean muscle mass from the control (Con) and ablated (Abl) legs divided by the body mass of the animals for n = 6 animals. * indicates significantly larger muscle mass than the controls.

After confirming a functional knockout of PTEN, the GTN or the TA muscle was removed from separate groups of mice to overload the PLN and SOL, or EDL muscle, respectively (Figure 4). Ablation of the synergists induced a significant hypertrophy in all of the muscles analysed (PLN  = 57.06±18.04%, SOL  = 38.26±15.27%, EDL  = 33.74±5.64%; p<0.05). However, there was no significant difference between the hypertrophy responses of the PTEN^−/−^ and PTEN^+/+^ animals (PLN  = 61.41±19.42%, SOL  = 50.53±12.79%, EDL  = 29.20±3.75% (p<0.05); Figure 4).

### Activation of Growth Signals Following Ablation

**Figure 5:**
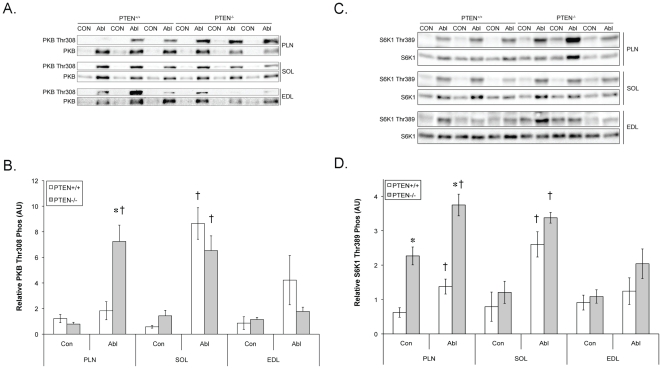
Muscle specific PTEN knockout alters PKB and S6K1 signalling in select muscles. The phosphorylation of PKB and S6K1 were determined in control muscles and muscles following overload of the plantaris (PLN) and soleus (SOL) muscles, by ablation of the gastrocnemius muscle for 10 days or the extensor digitorum longus (EDL) muscle by removal of the tibialis anterior muscle for 4 weeks. (A) Phosphorylation of PKB at Thr308 and its (B) quantification as well as (C) the phosphorylation of S6K1 at Thr389 and (D) its quantification. Data presented are mean ± SEM for n = 6 animals. * indicates significantly different than the wild type and † indicates significantly different than control.

Overload results in prolonged activation of the Class 1_a_ PI3K [40]. Thus we anticipated that ablation of PTEN would lead to even greater accumulation of PI(3,4,5)P_3_ and therefore enhanced PKB phosphorylation at the PI(3,4,5)P_3_ sensitive site, Thr^308^ (Figure 5). In the PLN of the PTEN^+/+^ mice, there was no net change in relative PKB Thr^308^ phosphorylation due to an increase in total PKB. However, in the PLN of PTEN^−/−^ animals the relative phosphorylation of PKB at Thr^308^ was significantly enhanced due to an increase in phosphorylation not a decrease in total protein (PTEN^+/+^ - Con PLN  = 1.21±0.32, Abl PLN = 1.83±0.70; PTEN^−/−^ - Con PLN  = 0.78±0.14, Abl PLN  = 7.24±1.28). The increase in PKB phosphorylation was similar in the EDL and SOL muscles of the PTEN^−/−^ and PTEN^+/+^ animals following ablation.

Thr^1462^ of TSC2 is known to be directly phosphorylated by PKB leading to the destabilisation of the TSC1/2 complex and activation of mTORC1 [41]. Therefore, we hypothesised that overload would lead to increased phosphorylation of S6K1 at the mTORC1 dependent site Thr^389^, a residue critical to S6K1 activation [42]. S6K1 Thr^389^ phosphorylation was higher in the control and overloaded PLN of PTEN^−/−^ mice (PTEN^+/+^- Con PLN  = 0.62±0.14, Abl PLN  = 1.37±0.21; PTEN^−/−^ - Con PLN  = 2.27±0.26, Abl PLN  = 3.75±0.31; Figure 5). However, the difference (delta) between the overloaded and control muscle was identical. There were no significant differences between groups for the other muscles.

### S6K1 Thr^389^ Phosphorylation Following Acute Resistance Exercise

**Figure 6:**
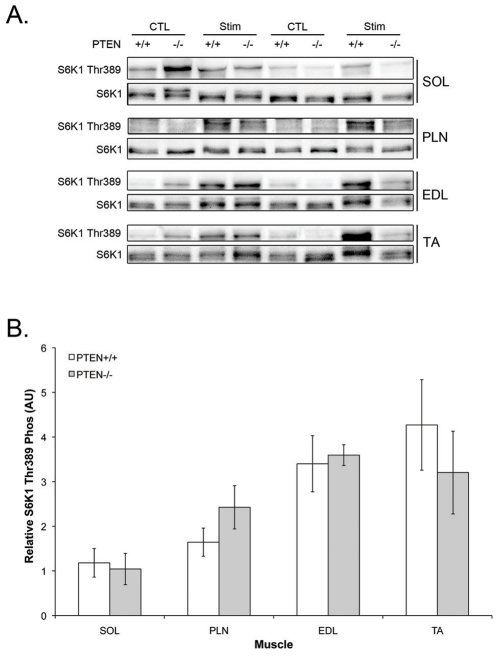
Muscle specific PTEN knockout has no effect of acute phosphorylation of S6K1. Wild type and PTEN^−/−^ mice underwent a 20-minute intermittent stimulation protocol to determine their acute response to resistance exercise. As observed previously, S6K phosphorylation was significantly higher in the TA and EDL muscles than the soleus and PLN. However, there was no difference in S6K1 activation between the wild type and PTEN^−/−^ mice. Data presented are mean ± SEM for n = 6 animals. * indicates significantly different than the control.

Acute resistance exercise is a more physiological model of muscle loading than synergist ablation. Therefore, the phosphorylation of S6K1 at Thr^389^ 1 hour after an acute bout of resistance exercise was determined in PTEN^+/+^ and PTEN^−/−^ mice. As previously reported [43], the change in phosphorylation of S6K1 was significantly higher in the TA and EDL than in the SOL with the PLN muscle seeing ∼50% of the activation seen in the TA and EDL (Figure 6). However, there was no significant difference in the phosphorylation of S6K1 between the PTEN^+/+^ and PTEN^−/−^ mice in any muscle analysed.

## Discussion

The present study demonstrates that resistance exercise does not alter the activity of the IGF-1 receptor or result in recruitment of PI3K to IRS1/2. Further, even though complete knockout of PTEN in muscle led to a reduction in insulin-stimulated phosphorylation of PKB in the EDL and increased wet mass in the TA and heart, the response to overload was unaltered. It is interesting to note that even though skeletal muscle is highly insulin sensitive and has a low incidence of transformation, PTEN is expressed at a substantially lower level in skeletal muscle than any other tissue analysed. These data suggest that growth factor signalling through PI(3,4,5)P_3_, and by extension PKB, does not play a significant role in normal skeletal muscle hypertrophy but may play a role in maintaining developmental growth and insulin sensitivity.

Insulin and insulin-like growth factor activation of mTORC1 is dependent on the activation of the IGF-1 receptor [44], the generation of PI(3,4,5)P_3_ at the membrane, and the subsequent activation of PKB [45], [46]. PI(3,4,5)P_3_ is primarily generated by the class IA phosphoinositide 3-kinases following recruitment to the membrane through the association of the regulatory p85 subunit with membrane-associated phosphotyrosine residues [47]. IRS1 and IRS2 are the predominant membrane-associated scaffolds for PI3K, with IRS1 associated p85 accounting for up to as much as 70% of total cellular PI3K activity [47]. We found no increase in IGF-1 receptor tyrosine phosphorylation or IRS1/2 associated p85 following resistance exercise and, from 30 minutes, p85 associated with IRS1/2 was reduced. As p85 can bind to other phosphoproteins [48] we also assessed phosphotyrosine associated p85 at 0 hr, 0.5 hrs and 18 hrs post stimulation and found no significant change in this measure. The increase in mTORC1 activity and PKB phosphorylation in the absence of IRS1/2 associated p85 is consistent with other models of contraction in which PKB is activated independent of any increase in tyrosine phosphorylation of the insulin receptor or IRS1 [49], [50]. Interestingly, O'Neil et al have shown that when mouse EDL muscles are stretched *ex vivo* for 60 minutes, mTORC1 is activated in a wortmannin insensitive manner [50], whereas Sakamoto et al. [49], demonstrated that the activation of PKB by endurance exercise is wortmannin sensitive. This indicates that exercise-induced accumulation of PI(3,4,5)P_3_ was required for the activation of PKB following some types of contraction. The absence of PI3K activation together with the requirement for PI(3,4,5,)P_3_ observed by Sakamoto suggested that inactivation of PTEN [51] could be important in contraction mediated PKB activation.

To determine whether PI(3,4,5)P_3_ accumulation was important for resistance exercise-induced signalling to mTORC1 we therefore sought to determine the effect of muscle-specific knockout of the 3-phosphatase PTEN. There are some disparities between the current data and previously published work on MCK-Cre^+/−^ PTEN^−/−^ mice. For instance, we observed a 10% increase in heart mass in the absence of PTEN compared to the ∼50% increase reported by Crackower et al [22]. Interestingly, the mass of the hearts in our control animals was approximately 10% larger than that reported by Crackower et al [22] suggesting that the disparity might be the result of differences in the strain background of the mice. The greatest difference between the current work and the literature is the substantially lower PTEN levels observed in muscle. Unlike Wijesekara et al, we found very little PTEN in wild type skeletal muscle [23]. In fact, compared to other organs, wild type muscle appeared to already be PTEN null. We confirmed the low levels of PTEN in muscle by western blot in rats and 5 other mouse strains, using 3 different antibodies and then validated these data with immunohistochemistry. Even though we looked at 5 mouse strains, it is still possible that the disparity in PTEN expression is the result of differences in the background strain of the transgenic line. There is evidence that the genetic background of a transgenic line can have a dramatic effect on phenotype [52]. For instance, altering the targeting subunit of the phosphatase PP1 leads to either an obese insulin resistant phenotype [53] or a normal phenotype depending on the donor background [54] even though both studies backcrossed onto the C57Bl/6 line. However, we believe that a more likely explanation could be the development of new antibodies that are more specific and technical differences in the histochemistry.

While the basal level of PTEN was lower than previously reported, the effect of PTEN knockout on insulin sensitivity was similar. Several previous reports have demonstrated a decrease in insulin sensitivity following loss of PTEN [23], [28]. Consistent with this, insulin-stimulated phosphorylation of PKB in the EDL was shifted to the right in the PTEN^−/−^ mice (Figure S2). Interestingly, the effect was not seen in the soleus muscle even though the basal expression of PTEN and the percent decrease in PTEN were greater. This suggests that fast skeletal muscle is more metabolically sensitive to loss of PTEN than slow muscle even though slow muscle has higher insulin sensitivity.

Since previous studies have shown that increasing PKB activity/expression leads to muscle growth in adult skeletal muscle [6], [7], we had expected that the growth response of muscle from PTEN null animals would have been greater due to greater PKB signalling. Indeed, we found significant increases in PKB Thr^308^ phosphorylation in the overloaded PLN. However, load induced growth was normal in PTEN null animals suggesting a dissociation between PI3K/PKB signalling and hypertrophy. This is likely because overload simultaneously activates AMPKα1 and AMPKα1 antagonises PKB phosphorylation of TSC2 [40], [55]. Therefore, while PKB phosphorylation is increased, there is no greater muscle growth. In support of this hypothesis, Mounier et al [56] showed that knocking out AMPKα1 leads to enhanced muscle growth following ablation. Therefore, activation of AMPKα1 during hypertrophy limits the activation of mTORC1 and reduces muscle growth. Furthermore, previous transgenic work has clearly demonstrated that AMPK can limit tumorigenesis in PTEN hypomorphic mice suggesting that a similar mechanism is at work during overload hypertrophy [57]. Crossing muscle specific PTEN knockouts with the AMPKα1 knockouts would allow us to directly test this hypothesis. However, there is evidence that the activation of AMPKα1 is not the only mechanism of action. Specifically, the fact that S6K1 Thr^389^ phosphorylation is elevated in the PLN in parallel with increased PKB Thr^308^ phosphorylation suggests that the inhibitory effect of AMPK on mTORC1 is not the only mechanism at work.

While the increase in PI3K/PKB activity in the overloaded PLN muscle did not lead to an increase in muscle growth, it could have led to alterations in phenotype that were not measured. During overload hypertrophy, there is an ∼19% increase in the capillary to fiber ratio in the first 15 days [58]. Plyley et al [58] suggested that the correlation between the increased fiber area and capillarisation are indicative of a common factor regulating both processes. Activation of PKB leads to an increase in the expression of VEGF and a resultant increase in capillary density and blood flow [59]. Therefore, while the increased activation of PKB in the overloaded PLN muscle of the PTEN^−/−^ mice did not enhance muscle growth, muscle blood flow may have been improved. Of note, there was only an increase in PKB phosphorylation in the overloaded muscle with the altered recruitment pattern (PLN muscle). Since changing recruitment could lead to greater metabolic stress and therefore greater angiogenesis, this finding is consistent with the hypothesis that the increase in PI3K/PKB activity is important in increasing blood flow to the muscle following overload.

In summary, loss of PTEN in skeletal muscle leads to an increase in the mass of selected muscles during development. However, the enhanced PI3K/PKB signalling following ablation does not lead to significant increases in muscle mass. This suggests that growth factor activation of PI3K/PKB plays a limited direct role in the hypertrophy of adult skeletal muscle.
